# Trends in Suicide by Level of Urbanization — United States, 1999–2015

**DOI:** 10.15585/mmwr.mm6610a2

**Published:** 2017-03-17

**Authors:** Scott R. Kegler, Deborah M. Stone, Kristin M. Holland

**Affiliations:** ^1^Division of Analysis, Research, and Practice Integration; ^2^Division of Violence Prevention, National Center for Injury Prevention and Control, CDC.

Suicide is a major and continuing public health concern in the United States. During 1999–2015, approximately 600,000 U.S. residents died by suicide, with the highest annual rate occurring in 2015 (*1*). Annual county-level mortality data from the National Vital Statistics System (NVSS) and annual county-level population data from the U.S. Census Bureau were used to analyze suicide rate trends during 1999–2015, with special emphasis on comparing more urban and less urban areas. U.S. counties were grouped by level of urbanization using a six-level classification scheme. To evaluate rate trends, joinpoint regression methodology was applied to the time-series data for each level of urbanization. Suicide rates significantly increased over the study period for all county groupings and accelerated significantly in 2007–2008 for the medium metro, small metro, and non-metro groupings. Understanding suicide trends by urbanization level can help identify geographic areas of highest risk and focus prevention efforts. Communities can benefit from implementing policies, programs, and practices based on the best available evidence regarding suicide prevention and key risk factors. Many approaches are applicable regardless of urbanization level, whereas certain strategies might be particularly relevant in less urban areas affected by difficult economic conditions, limited access to helping services, and social isolation.

NVSS county-level mortality data for 1999–2015 were used to identify suicides among U.S. residents (excluding those aged <10 years because intent for self-harm typically is not attributed to young children) based on the *International Classification of Diseases, 10th Revision* (ICD-10) underlying cause codes X60–X84, Y87.0, and U03. Annual suicide counts were tabulated for county groupings defined according to a six-level urbanization classification scheme employed in the CDC WONDER reporting application (*2*). This classification scheme represents the level of urbanization as of 2006, selected to coincide with the middle of the study period. From most urban to least urban, the county classifications are large central metro, large fringe metro, medium metro, small metro, micropolitan (i.e., town/city; non-metro), and non-core (i.e., rural; non-metro).* Tabulated counts were combined with U.S. Census Bureau annual county-level population estimates to calculate annual suicide rates (per 100,000 residents aged ≥10 years). Rates were age-adjusted to the year 2000 U.S. standard.

Trends were evaluated by applying joinpoint regression methodology^†^ to the annual suicide rate time series for each county grouping. This modeling approach simultaneously identifies statistically significant trends as well as shifts in trends that occur within a time series. Based on the results of the modeling process, the study frame was subsequently divided into an earlier 9-year period (1999–2007) and a later 8-year period (2008–2015) for purposes of examining changes in suicide rates by other factors, including sex, age group, race/ethnicity, and method of suicide.

Increases in annual suicide rates over the study period occurred among all six county urbanization classifications (Figure). Rates at the beginning of the study period were lowest for the more urban counties and highest for the less urban counties, a gap that widened over time. The joinpoint regression results supported the same general conclusions, but further suggested that the gap in rates widened most conspicuously after 2007–2008 (Table 1) (Table 2). For the large central metro and large fringe metro county groupings, the joinpoint modeling process identified continuous and statistically significant rate increases over the entire study period (Table 1). For the medium metro, small metro, micropolitan, and non-core county groupings, statistically significant rate increases were also identified over the earlier part of the study period; modeled rate increases for these four county groupings accelerated significantly in 2007–2008 (Table 2).

**FIGURE:**
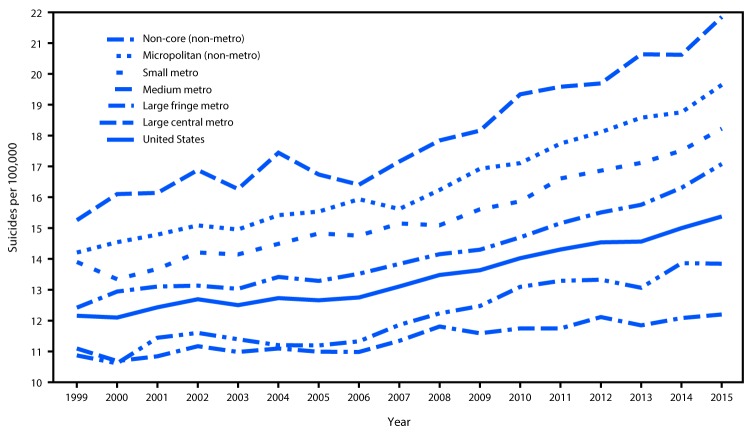
Suicide rates* by level of county urbanization^†^ — United States, 1999–2015 * Per 100,000 residents aged ≥10 years, age-adjusted to the year 2000 U.S. standard. ^†^ The six classification levels for counties were 1) large central metro: part of a metropolitan statistical area with ≥1 million population and covers a principal city; 2) large fringe metro: part of a metropolitan statistical area with ≥1 million population but does not cover a principal city; 3) medium metro: part of a metropolitan statistical area with ≥250,000 but <1 million population; 4) small metro: part of a metropolitan statistical area with <250,000 population; 5) micropolitan (non-metro): part of a micropolitan statistical area (has an urban cluster of ≥10,000 but <50,000 population); and 6) non-core (non-metro): not part of a metropolitan or micropolitan statistical area.

**TABLE 1:** Trends in suicide rates by large county level of urbanization* — United States, 1999–2015

County urbanization level	No. of counties	No. of suicides	Overall annual suicide rate increase^†^	p-value	Joinpoint year
Large central metro	63	150,636	0.09	<0.01	—
Large fringe metro	352	133,479	0.20	<0.01	—

* Counties or county-equivalents; a small number of counties were combined into multicounty groupings. The six classification levels for counties were 1) large central metro: part of a metropolitan statistical area with ≥1 million population and covers a principal city; 2) large fringe metro: part of a metropolitan statistical area with ≥1 million population but does not cover a principal city; 3) medium metro: part of a metropolitan statistical area with ≥250,000 but <1 million population; 4) small metro: part of a metropolitan statistical area with <250,000 population; 5) micropolitan (non-metro): part of a micropolitan statistical area (has an urban cluster of ≥10,000 but <50,000 population); and 6) non-core (non-metro): not part of a metropolitan or micropolitan statistical area.

^†^ Per 100,000 residents aged ≥10 years, age-adjusted to the year 2000 U.S. standard.

**TABLE 2:** Trends in suicide rates by medium and small county level of urbanization* — United States, 1999–2015

County urbanization level	No. of counties	No. of suicides	Initial annual suicide rate increase^†^	p-value	Joinpoint year	Annual suicide rate increase^†^ after joinpoint year	p-value for difference
Medium metro	331	126,447	0.14	<0.01	2008	0.41	<0.01
Small metro	339	64,739	0.19	<0.01	2008	0.41	<0.01
Micropolitan (non-metro)	694	75,002	0.19	<0.01	2007	0.45	<0.01
Non-core (non-metro)	1,355	52,075	0.18	<0.05	2007	0.55	<0.01

* Counties or county-equivalents; a small number of counties were combined into multicounty groupings. The six classification levels for counties were 1) large central metro: part of a metropolitan statistical area with ≥1 million population and covers a principal city; 2) large fringe metro: part of a metropolitan statistical area with ≥1 million population but does not cover a principal city; 3) medium metro: part of a metropolitan statistical area with ≥250,000 but <1 million population; 4) small metro: part of a metropolitan statistical area with <250,000 population; 5) micropolitan (non-metro): part of a micropolitan statistical area (has an urban cluster of ≥10,000 but <50,000 population); and 6) non-core (non-metro): not part of a metropolitan or micropolitan statistical area.

^†^ Per 100,000 residents aged ≥10 years, age-adjusted to the year 2000 U.S. standard.

During both 1999–2007 and 2008–2015, overall rates of suicide among males were approximately four times those among females; rates increased across the two periods for both males (from 21.1 per 100,000 to 23.3) and females (from 5.0 to 6.2) (Table 3). By age group, the highest rates were among persons aged 35–64 years and ≥75 years; the 35–64 year age group also showed the largest rate increase (from 14.9 to 17.9). By race/ethnicity, non-Hispanic whites and American Indian/Alaska Natives had the highest rates of suicide, with rates for both groups showing notable increases across periods (from 14.9 to 18.1 and from 15.8 to 20.0, respectively). Rates among non-Hispanic blacks and Asian/Pacific Islanders and among Hispanics were much lower, and showed comparatively modest increases across periods. Rates increased across periods for both non-firearm and firearm suicide, with a greater increase in the rate of non-firearm suicide, particularly from suffocation (which includes hanging).

**TABLE 3:** Average annual suicide rates,* overall and by sex, age group, race/ethnicity, and suicide method — United States, 1999–2007 and 2008–2015

Characteristic	Period	
	1999–2007	2008–2015
**Overall^†^**	**12.6**	**14.4**
**Sex^†^**		
Male	21.1	23.3
Female	5.0	6.2
**Age group (yrs)**		
10–19	4.3	4.9
20–34	12.6	14.1
35–64	14.9	17.9
65–74	12.7	14.4
≥75	17.2	17.0
**Race/Ethnicity^†,§^**		
White, non-Hispanic	14.9	18.1
Black, non-Hispanic	6.3	6.5
American Indian/Alaska Native, non-Hispanic	15.8	20.0
Asian/Pacific Islander, non-Hispanic	6.5	7.0
Hispanic	6.7	6.8
**Method^†^**		
Firearm	6.7	7.2
Non-firearm	5.9	7.2
Suffocation (including hanging)	2.7	3.7
Poisoning	2.2	2.4
Drug	1.6	1.9
Non-drug	0.6	0.5
Other non-firearm	1.0	1.1

* Per 100,000 residents aged ≥10 years.

^†^ Age-adjusted to the year 2000 U.S. standard.

^§^ Hispanic persons might be of any race.

## Discussion

After declining since 1986, the U.S. suicide rate increased during 2000–2015 (*3*). This study provides added support to previous findings that a geographic disparity in suicide rates exists in the United States, with higher rates in less urban areas and lower rates in more urban areas (*4*) and extends these findings to characterize suicide trends by urbanization level over time. Specifically, the current study found that suicide rates across all urbanization levels increased during the period 1999–2015, the gap in rates between less urban and more urban areas widened over time, and rates in medium metro, small metro, and non-metro areas increased at a more rapid pace beginning in 2007–2008.

Geographic disparities in suicide rates might be associated with suicide risk factors known to be highly prevalent in less urban areas, such as limited access to mental health care, made worse by shortages in behavioral health care providers in these areas (*5*), and greater social isolation (*5,6*). Such disparities might also reflect the influence of the opioid overdose epidemic. This epidemic is known to have disproportionately affected less urban areas during the earlier part of the study period (*7*), and opioid misuse is associated with increased risk for suicide (*8*). That increases in suicide rates outside large metro areas accelerated in 2007–2008 might reflect the influence of the economic recession of 2007–2009, which had a disproportionate impact and involved longer recovery times in less urban areas (*9*). The potential cumulative burden of suicide risk factors in less urban areas might affect not only individuals but relationships, families, and communities as well, suggesting the need for comprehensive suicide prevention measures. Given the disparate nature of suicide risk factors beyond mental health factors alone (e.g., social isolation, financial hardship, and access to lethal means), and the far-reaching emotional and economic consequences of suicide on families and communities, implementing such measures calls for a broad public health approach at the individual, community, and societal levels.

Just as suicide is not caused by a single factor, research suggests that suicide prevention cannot be achieved with a single strategy. Suicide prevention efforts might be most effective when multiple strategies operating across the range of contexts in which persons live and work are combined (*10*). Many prevention strategies and approaches might be broadly applicable for all communities regardless of size, whereas others might be particularly relevant for less urban areas. For example, all communities might benefit from strategies that enhance coping and problem-solving skills, strengthen economic support during times of financial hardship, and identify and support persons at risk for suicide (e.g., through gatekeeper training, crisis intervention, and effective treatments). Reducing access to lethal means among persons at risk, improving organizational policies and culture to promote positive social norms such as help-seeking, supporting surviving friends and family members, and promoting safe messaging and news reporting about suicide to prevent suicide contagion are additional strategies that might benefit all communities (*10*). On the other hand, residents in less urban areas might benefit particularly from prevention strategies that address provider shortages, for example, through programs that incentivize mental health clinicians to work in underserved areas, or through the provision of treatment via telephone, video, and web-based technologies. Less urban areas also might benefit from suicide prevention strategies that promote social connectedness through community engagement activities that provide residents with the opportunity to interact with each other and to become familiar with supportive organizations and resources (*10*).

The findings in this report are subject to at least two limitations. First, a small fraction of suicide records (<0.4%) were excluded from the analysis because of missing ethnicity data, resulting in a slight downward bias on some rate estimates. Second, individual counties were considered to embody the same level of urbanization throughout the 1999–2015 study period; the year 2006 urbanization classification scheme does not reflect changes in county composition over time. However, an earlier comparison of the year 2006 classification scheme with an updated 2013 classification scheme indicates that >90% of counties retained the same status and that when a change in classification occurred, it typically involved a shift to an adjacent level of urbanization; the potential influence of the constant classification scheme should therefore be relatively minimal.

The current study highlights higher rates of suicide in areas with lower levels of urbanization, and demonstrates a growing disparity between rates in less urban and more urban areas of the United States. Suicide is preventable, and evidence-based strategies to prevent suicide in both less urban and more urban areas exist. Resources such as CDC’s *Preventing Suicide: a Technical Package of Policies, Programs, and Practices* (*10*) and the National Violent Death Reporting System can help states and communities prioritize prevention efforts and address persistent upward trends in suicide rates.

**Summary**

**What is already known about this topic?**

The U.S. suicide rate has been increasing since 2000. Rates in less urban areas have been higher than rates in more urban areas, with some evidence of a growing difference.

**What is added by this report?**

During 1999–2015, suicide rates increased across all levels of urbanization, with the gap in rates between less urban and more urban areas widening over time, most conspicuously over the later part of this period. Geographic disparities in suicide rates might reflect suicide risk factors known to be prevalent in less urban areas, such as limited access to mental health care, social isolation, and the opioid overdose epidemic, because opioid misuse is associated with increased risk for suicide. That the gap in rates began to widen more noticeably after 2007–2008 might reflect the influence of the economic recession, which disproportionately affected less urban areas.

**What are the implications for public health practice?**

There is a growing need for comprehensive suicide prevention employing a broad public health approach. This might include strategies applicable for all communities (e.g., strengthening economic support during times of financial hardship and teaching coping and problem-solving skills) along with strategies that address subsets of the population at increased risk, such as rural communities (e.g., programs that address provider shortages and promote social connectedness). CDC’s technical package of multisector policies, programs, and practices serves as a resource for states and communities to guide decision-making based on the best available evidence for preventing suicide.

## Footnotes

* The six classification levels for counties are 1) large central metro: part of a metropolitan statistical area with ≥1 million population and covers a principal city; 2) large fringe metro: part of a metropolitan statistical area with ≥1 million population but does not cover a principal city; 3) medium metro: part of a metropolitan statistical area with ≥250,000 but <1 million population; 4) small metro: part of a metropolitan statistical area with <250,000 population; 5) micropolitan (non-metro): part of a micropolitan statistical area (has an urban cluster of ≥10,000 but <50,000 population); and 6) non-core (non-metro): not part of a metropolitan or micropolitan statistical area.

† https://surveillance.cancer.gov/joinpoint/.
